# Metabolic Evaluation of Epilepsy: A Diagnostic Algorithm With Focus on Treatable Conditions

**DOI:** 10.3389/fneur.2018.01016

**Published:** 2018-12-03

**Authors:** Clara D. M. van Karnebeek, Bryan Sayson, Jessica J. Y. Lee, Laura A. Tseng, Nenad Blau, Gabriella A. Horvath, Carlos R. Ferreira

**Affiliations:** ^1^Department of Pediatrics, University of British Columbia, Vancouver, BC, Canada; ^2^Centre for Molecular Medicine and Therapeutics, BC Children's Hospital Research Institute, University of British Columbia, Vancouver, BC, Canada; ^3^Departments of Pediatrics and Clinical Genetics, Emma Children's Hospital, Amsterdam University Medical Centres, Amsterdam, Netherlands; ^4^Division of Biochemical Diseases, Department of Pediatrics, BC Children's Hospital Research Institute, University of British Columbia, Vancouver, BC, Canada; ^5^Dietmar-Hopp Metabolic Center, University Children's Hospital, Heidelberg, Germany; ^6^Division of Metabolism, University Children's Hospital, Zurich, Switzerland; ^7^Division of Genetics and Metabolism, Children's National Health System, Washington, DC, United States; ^8^National Human Genome Research Institute, National Institutes of Health, Bethesda, MD, United States

**Keywords:** inborn errors of metabolism, metabolic epilepsy, seizures, diagnostic algorithm, treatment

## Abstract

Although inborn errors of metabolism do not represent the most common cause of seizures, their early identification is of utmost importance, since many will require therapeutic measures beyond that of common anti-epileptic drugs, either in order to control seizures, or to decrease the risk of neurodegeneration. We translate the currently-known literature on metabolic etiologies of epilepsy (268 inborn errors of metabolism belonging to 21 categories, with 74 treatable errors), into a 2-tiered diagnostic algorithm, with the first-tier comprising accessible, affordable, and less invasive screening tests in urine and blood, with the potential to identify the majority of treatable conditions, while the second-tier tests are ordered based on individual clinical signs and symptoms. This resource aims to support the pediatrician, neurologist, biochemical, and clinical geneticists in early identification of treatable inborn errors of metabolism in a child with seizures, allowing for timely initiation of targeted therapy with the potential to improve outcomes.

## Introduction

The occurrence of epilepsy in the general population is relatively common. Just in the United States, it affects 1.2% of the population, with 3 million adults and 470,000 children suffering from it (1). In cases of neonatal seizures, inborn errors of metabolism (IEMs) account for 1.1% (2) −7.4% (3) of all cases. Although IEMs are responsible for only a fraction of these cases, it is imperative to identify them, as there is often treatment available which can mitigate or even prevent major neurological sequelae (4). Elucidating the etiology of a seizure disorder can also prompt the investigation of extra-neurologic systems commonly affected by IEMs (i.e., arrhythmias in mitochondrial disease, liver disease, hearing or retinal involvement in peroxisomal disorders, etc.). It is important to consider the possibility of an IEM in a patient presenting with seizures of an unknown cause not only due to the amenability to causal treatment of the seizures and the possible co-morbidities, but also in order to adequately perform genetic counseling and to more accurately predict the disease trajectory and prognosis. Finally, a timely diagnosis will not only avoid unnecessary delay and burden to the patient, it might also prevent failed attempts to control seizure with standard antiepileptic drugs, often not without side effects, and it has the potential to decrease the number and cost of unnecessary tests.

Even when seizures are the major presenting feature of an IEM, such as in pyridoxine-dependent epilepsy (PDE), diagnosis is often difficult due to the broad differential diagnoses varying from environmental to non-metabolic genetic etiologies (e.g., hypoxic-ischemic encephalopathy or genetic channelopathy) (5). In addition, there is a great number of metabolic epilepsies, posing a considerable challenge in identification based upon primary symptomology; hence, standardized screening for a subset for IEMs is advisable. Finally, the clinical picture even for a specific metabolic condition may be often complex and mask an otherwise evident IEM. An example is PDE, which can present in the neonatal period not only with an epileptic encephalopathy but also with metabolic acidosis, electrolyte disturbances, abdominal distension mimicking sepsis, or structural brain anomalies that might be considered a sufficient explanation for seizures (5).

Patients with early-onset epilepsy can be evaluated in several different departments within a given hospital, which necessitates a standardized testing protocol in order to facilitate effective coordination of the various departments, as well as to avoid unnecessary diagnostic delay. Standardized testing will prevent the administration of repeat tests, increasing the efficacy of laboratory resources as well as diminishing cost. Finally, the use of a standardized approach will avoid missed diagnoses, especially for clinicians with less experience.

An indirect benefit from establishing an early diagnosis stems from the fact that increased awareness of these IEMs can further expand the knowledge base and clinical phenotype. For example, pyridox(am)ine 5′-phosphate oxidase (PNPO) deficiency has, in the past, been reported to have high mortality in the few cases in which it was reported. However, awareness and subsequent prompt diagnosis has increased survivability as well as expanded the phenotype (6).

## Methods

Given the multitude of aforementioned benefits from establishing an early diagnosis of IEMs, we propose a diagnostic approach in patients with epilepsy, with emphasis on early identification of those amenable to treatment. The algorithm is structured according to the TIDE protocol for diagnosis of treatable intellectual disabilities (7, 8). All metabolic conditions included in the IEMbase tool [www.IEMbase.org; (9)] and in the proposed Nosology (10) were individually searched along with the terms “seizures,” “epilepsy,” or “convulsions” in Pubmed (1966-December 2017) to identify all IEMs for which epilepsy is a feature, and for which therapy targeting the underlying pathophysiology is available and deemed at least partially effective or preventive, with sufficient evidence (level IV or higher according to the Center for Evidence-Based Medicine (CEBM).

## Results

Our systematic review identified 268 IEMs with epilepsy as a primary or secondary feature, listed in Supplementary Table 1 following the nomenclature of IEMs of the recently-proposed Nosology (10). Of these, 74 conditions are treatable. These IEMs include disorders of amino acids (*n* = 43, 12 treatable); sterols and bile acids (*n* = 7, 1 of which is treatable); creatine (*n* = 3, all treatable); fatty aldehydes (*n* = 1, not treatable); fatty acids, carnitine and ketones (*n* = 9, 6 treatable); complex lipids (*n* = 8, none treatable); carbohydrates and polyols (*n* = 8, half of them treatable); lysosomes (*n* = 23, none treatable); lipofuscin storage (*n* = 9, 1 treatable); metals (*n* = 5, 3 treatable); mitochondria (*n* = 26, 7 treatable); autophagy (*n* = 2, neither of which is treatable); neurotransmitters (*n* = 4, all treatable); organic acids (*n* = 10, 6 treatable); peroxisomes (*n* = 7, 1 treatable); purines and pyrimidines (*n* = 5, 2 treatable); urea cycle (*n* = 9, all treatable); vitamins/co-factors (*n* = 23, 12 treatable); glutathione (*n* = 1, not treatable), heme (*n* = 2, 1 treatable); and glycans and glycolipids (*n* = 63, 1 treatable). The therapeutic modalities available for these IEMs include: “sick–day” management, medical diets, cofactor/vitamin supplements; substrate inhibition, stem cell transplant, and gene therapy. Therapeutic effects vary from complete control, improvement or prevention of epilepsy both clinically and on EEG. Secondary outcomes include improvement and/or stabilization of psychomotor/cognitive development; behavior/psychiatric disturbances; seizures; and neurologic and systemic manifestations (11). Supplementary Table 1 provides more information on these IEMs, among others whether or not included in newborn screening panels.

### When to suspect metabolic epilepsies

The presence of any of the following features should raise suspicion for a metabolic etiology of epilepsy (12):

- Dysmorphic features (e.g., peroxisomal disorders, CDGs, crotonase deficiency)
- Organomegaly
- Positive family history for similar condition or death of unknown etiology
- Parental consanguinity
- Developmental regression after a period of apparent normalcy
- Fluctuating course of illness
- Ophthalmological problems (cataracts, retinitis pigmentosa, cherry red spot, optic nerve atrophy)
- High-anion gap metabolic acidosis
- Neonatal ketonuria
- Unusual body fluid odor
- Seizures worsening with fasting (GLUT1) or with high protein meals (urea cycle defects)
- Seizure worsening with anti-epileptic drugs
- Progressive myoclonic epilepsy phenotype in adolescence or young adulthood.

The age of presentation can be a diagnostic indication of the form IEM affecting the patient. Therefore, it is practical to organize epilepsies caused by IEM according to age, which results in a distribution of epilepsies occurring in the neonatal or infantile period, in early childhood, or those that present in late childhood and adolescence. Table 1 outlines this organization. This information may be useful in ordering Tier 2 testing.

**Table 1:** Metabolic epilepsies classified according to age of onset.

**NEONATAL-INFANTILE ONSET**
Urea cycle disorders*
Maple syrup urine disease*
Organic acidemias*
Hepatocardiomuscular carnitine palmitoyltransferase II deficiency
Cobalamin C disease*
MTHFR deficiency*
Pyridoxine-dependent epilepsy*
Pyridox(am)ine 5'-phosphate oxidase deficiency*
PROSC deficiency*
Folinic acid responsive epilepsy*
GLUT1 deficiency*
Biotinidase deficiency*
Holocarboxylase synthetase deficiency*
Severe classic nonketotic hyperglycinemia
Serine biosynthesis defects*
Glutamine synthetase deficiency
Asparagine synthetase deficiency
Molybdenum cofactor deficiency
Isolated sulfite oxidase deficiency
Fumarate hydratase deficiency
Adenylosuccinate lyase deficiency type 1
GABA transaminase deficiency
Menkes disease*
Pyruvate dehydrogenase deficiency
Pyruvate carboxylase deficiency type A
Mitochondrial respiratory chain defects
Mitochondrial thiamine pyrophosphate transporter deficiency
Congenital disorders of glycosylation
Peroxisomal biogenesis disorders and D-bifunctional protein deficiency
Sialidosis type 2
Gaucher disease type 2
Prosaposin deficiency
Classic infantile GM1 gangliosidosis
Glycolipid synthesis deficiency (GM3 synthase and ST3GAL3 deficiencies)
Hyperinsulinism-hyperammonemia syndrome*
Disorders of lipoic acid synthesis
Mitochondrial glutamate transporter deficiency
Dihydrofolate reductase deficiency*
Glycosylphosphatidylinositol biosynthesis defects
Hypophosphatasia*
**LATE INFANTILE**
Creatine synthesis defects*
Organic acidemias*
Juvenile GM1 gangliosidosis
GM2 gangliosidosis
Classic late infantile neuronal ceroid lipofuscinosis*
Succinic semialdehyde dehydrogenase deficiency
CAD trifunctional protein deficiency*
GLUT1 deficiency*
Disorders of monoamine metabolism (AADC deficiency)*
Disorders of tetrahydrobiopterin metabolism (PTPS and DHPR deficiencies)*
Mitochondrial respiratory chain defects
Metachromatic leukodystrophy
Biotin-thiamine-responsive basal ganglia disease*
Congenital disorders of glycosylation
Folate receptor deficiency*
Dihydrofolate reductase deficiency*
Vici syndrome
Milder variants of PDE or PNPO deficiency*
Attenuated classic nonketotic hyperglycinemia*
**LATE CHILDHOOD-ADOLESCENCE**
Sialidosis type 1
Juvenile neuronal ceroid lipofuscinosis
Mitochondrial encephalomyopathy, lactic acidosis, and stroke-like episodes (MELAS)
Myoclonic epilepsy with ragged-red fibers (MERRF)
Gaucher disease type 3
Niemann-Pick type C
X-linked adrenoleukodystrophy*
Primary CoQ deficiency*
Wilson disease*
Porphyria*
Aspartylglucosaminuria
Salla disease

*Asterisks indicate treatable conditions*.

The particular type of seizure can occasionally raise suspicion for a specific disorder. For example, epilepsia partialis continua can be the first clue toward a diagnosis of a POLG-related disorder (13), patients with certain forms of congenital disorders of glycosylation can present with migrating partial seizures of infancy (14), while patients with guanidinoacetate methyltransferase deficiency can have drop attacks and Lennox-Gastaut syndrome (15). For other conditions, the evolution of seizure over time have been well defined, such as for Menkes disease, in which pattern of seizures can be divided in three stages: an early stage at around 3 months showing focal clonic status epilepticus, an intermediate stage at about 10 months showing intractable infantile spasms, and a late stage at about 25 months showing multifocal seizures, tonic spasms and myoclonus (16). In the majority of metabolic epilepsies, however, the particular etiology of seizures cannot be predicted from its semiology. A perfect example of this is that of GLUT1 deficiency, in which the type of seizures is quite variable, being mixed in 68%, generalized tonic-clonic in 53%, absence in 49%, complex partial in 37%, myoclonic in 27%, drop attacks in 26%, tonic in 12%, simple partial in 3%, and spasms in 3% (17).

Although many IEMs will manifest with epilepsy refractory to multiple standard anti-epileptic drugs (AEDs), a favorable response to these drugs does not rule out metabolic epilepsies. For example, patients with GLUT1 deficiency can show response to standard AEDs (17, 18), and patients with PDE can also show some response (5), or can even show lack of seizures after a period of time without pyridoxine supplementation (19). On the other hand, the lack of response to standard treatment for these conditions can also not be used as a criteria to rule out the diagnosis: patients with GLUT1 deficiency can still show recurrence of seizures despite adequate ketosis (20), and in fact up to one third of patients respond poorly to a ketogenic diet (21), while patients with PDE might not show an instant and obvious response to pyridoxine administration (5).

Finally, the presence of specific electroencephalographic (EEG) or brain neuroimaging findings can also provide important clues toward the right diagnosis. Some IEMs can have characteristic EEG changes, such as the comb-like rhythm seen in patients with maple syrup urine disease (22). Certain IEMs can be accompanied by structural brain anomalies identifiable by brain MRI, such as a dysgenetic corpus callosum seen commonly in patients with pyruvate dehydrogenase complex deficiency or glycine encephalopathy (23). Brain magnetic resonance spectroscopy can sometimes point toward the right diagnosis, as has been reported in patients with GABA transaminase deficiency (24), fatty acid oxidation disorders (25), mitochondrial disease or glycine encephalopathy (26). Table 2 provides a summary of the salient clinical, laboratory, electrographic, and neuroimaging finding of the most common metabolic epilepsies.

**Table 2:** Summary of clinical, laboratory, EEG, and neuroimaging finding of metabolic epilepsy.

**IEM**	**Neurologic**	**Non-neurologic**	**Laboratory**	**EEG**	**Brain MRI**	**Brain MRS**
Urea cycle disorders	Encephalopathy	Liver disease (sometimes)	Hyperammonemia. Respiratory alkalosis. Increased glutamine.	Slow background	Cortical and subcortical edema. BG T2-hyperintensity with thalamic sparing. Scalloped ribbon of DWI restriction at insular gray-white interface.	Prominent Glx peak
Organic acidemias	Encephalopathy. Choreoathetosis.	Cytopenias. Pancreatitis. Cardiomyopathy (PA). Renal disease (MMA).	Hyperammonemia. High-anion gap metabolic acidosis. Ketotic hyperglycinemia.	Slow background. Burst-suppression possible.	Diffuse swelling neonatally; delayed myelination and globi pallidi lesions later.	Decreased Glx peak (PA)
Disorders of biotin metabolism	Encephalopathy	Erythroderma or ichthyosis.	Hyperammonemia. High-anion gap metabolic acidosis. Lactic acidosis. Ketosis.	Burst-suppression	Intraventricular hemorrhage. Subependymal cysts.	Lactate peak
MSUD	Encephalopathy; opistothonus; bicycling/fencing movements.	Sweet (“maple syrup”) smell	Ketosis. Hypernatremia. Increased BCAAs and BCKAs.	Comb-like rhythm	Increased signal and cytotoxic edema myelinated structures, vasogenic edema of unmyelinated tracts	BCAA/BCKA peak (0.9 ppm)
Fatty acid oxidation defects	Encephalopathy (“Reye syndrome”)	Lipid storage myopathy. Liver disease. Renal cysts (GA2).	Hypoketotic hypoglycemia	Slow background	T2 hyperintensities in periventricular and subcortical WM (GA2)	Lipid peak (0.9 and 1.3 ppm)
Primary lactic acidosis	Encephalopathy. Infantile Parkinsonism (PC deficiency).	Dysmorphic features (PDH deficiency)	Lactic acidosis	Slow background, multifocal spikes.	T2 hyperintensities and DWI restriction of dorsal brainstem, cerebral peduncles, corticospinal tracts; subependymal cysts.	Lactate peak
Glycine encephalopathy	Seizures	None	High CSF glycine and CSF/plasma glycine ratio	Burst-suppression	Dysgenesis of the CC. T2 hyperintensities and DWI restriction of myelinated tracts	Glycine peak (3.55 ppm)
Molybdenum cofactor/sulfite oxidase deficiency	Seizures. Hyperekplexia.	None	Elevated S-sulfocysteine; low cysteine, high taurine. Increased AASA and pipecolic acid.	Burst-suppression	Diffuse swelling followed by cystic changes	S-sulfocysteine peak (3.61 ppm); taurine peak (3.24 and 3.42 ppm)
Disorders of GABA metabolism	Seizures. Hypersomnolence. Choreoathetosis.	Overgrowth (GABAT)	Elevated urine 4-hydroxybutyric acid (SSADH); elevated GABA, beta-alanine and homocarnosine (GABAT)	Slow background, multifocal spikes, burst-suppression.	T2 hyperintensities of globi pallidi, dentate and subthalamic nucleus (SSADH)	GABA peak (2.2–2.4 ppm; GABAT)
PDE	Seizures	None	Increased AASA and pipecolic acid	Slow background, multifocal spikes, burst-suppression.	Usually normal; can have dysgenetic CC	Decreased NAA peak (over time)
Serine biosynthesis disorders	Microcephaly. Seizures.	Ichthyosis. Ectropion, eclabion (Neu-Laxova)	Low serine in plasma and CSF	Multifocal spikes; hypsarrhythmia.	Hypomyelination	Decreased NAA peak; increased choline peak
Lysosomal storage disorders	Neurodegeneration.	Hydrops fetalis. Dermal melanosis. Ichthyosis (Gaucher type 2).	Decrease in specific enzyme activity. Vacuolated lymphocytes (CLN3 disease)	Fast central spikes (Tay-Sachs); vertex sharp waves (sialidosis)	Hypomyelination (GM1 and GM2 gangliosidosis, fucosidosis, Salla disease). Subdural fluid collections (NCLs).	Broad peak centered around 3.7 ppm
Peroxisonal disorders	Hypotonia. Seizures.	Cholestasis; renal cysts; epiphyseal stippling. Dysmorphic features.	Elevated VLCFA, phytanic acid, bile acid intermediates, pipecolic acid, low plasmalogens,	Multifocal spikes; hypsarrhythmia.	Perisylvian polymicrogyria and pachygyria; hypomyelination; subependymal cysts.	Lipid peak (0.9 and 1.3 ppm)
Congenital disorders of glycosylation	Hypotonia. Seizures.	Inverted nipples. Abnormal fat pads.	Elevated transaminases; coagulopathy; endocrine abnormalities	Multifocal epileptic discharges.	Pontocerebellar hypoplasia.	Decreased NAA peak
Disorders of copper metabolism	Seizures	Pili torti. Cutis laxa. Bladder divderticula. Metaphyseal lesions. Wormian bones.	Low serum copper and ceruloplasmin; high urine copper.	Burst-suppression	Arterial tortuosity. Subdural collections.	Decreased NAA peak
GLUT1 deficiency	Seizures. Abnormal eye movements.	Hemolytic anemia, pseudohyperkalemia, cataracts (specific mutations)	Low CSF glucose and lactate; low CSF/serum glucose ratio	Variable depending on type of seizure	Normal	Normal

*7DHC, 7-dehydrocholesterol; AASA, alpha-aminoadipic semialdehyde; BCAAs, branched-chain amino acids; BCKAs, branched-chain ketoacids; BGF, basal ganglia; CC, corpus callosum; DWI, diffusion weighted imaging; GA2, glutaric aciduria type 2; GABAT, GABA transaminase; Glx, glutamine/glutamate; MSUD, maple syrup urine disease; NAA, N-acetylaspartate; NCLs, neuronal ceroid lipofuscinosis; PA, propionic acidemia; PC, pyruvate carboxylase; PDE, pyridoxine-dependent epilepsy; PDH, pyruvate dehydrogenase; WM, white matter*.

### Metabolic investigational algorithm for patients with idiopathic or early onset epilepsy

#### Tier 1: initial screening in all patients

We propose that the following initial screening investigations be carried out for patients in whom epilepsy remains a prominent feature of unknown cause. The tests included in the 1st tier: (a) have the potential to identify 2 or more treatable metabolic epilepsies; (b) are accessible via standard clinical chemistry and biochemical genetics laboratories; (c) are relatively affordable and covered by insurance; and (d) are non-invasive, requiring only blood or dried blood spots and urine collection. Refer to Table 3 for test included in the first tier, and the abnormalities that can be found in those tests in different IEMs.

**Table 3:** IEMs identified by each of the Tier 1 diagnostic tests.

**Source**	**Diagnostic Test**	**Related IEM**
BLOOD:	Comprehensive metabolic panel	Glucose (low in FAODs and HIHA)
		Anion gap (elevated in organic acidemias)
		Liver transaminases (elevated in CDGs and mitochondrial depletion syndromes)
		Alkaline phosphatase (low in hypophosphatasia, elevated in GPI biosynthesis defects)
	Blood gases	Organic acidemias (low pH)
		Urea cycle disorders (high pH)
	Ammonia	Urea cycle disorders
		Organic acidemias
		HIHA
		HHH syndrome
		Lysinuric protein intolerance
		Pyruvate carboxylase deficiency
	Creatine kinase	FAODs
		Dystroglycanopathy type-CDG
	Uric acid	Molybdenum cofactor deficiency (low)
	Lactate/pyruvate	PDH deficiency
		Pyruvate carboxylase deficiency
		Biotinidase deficiency
		Mitochondrial respiratory chain defects
		Lipoic acid synthesis defects
	Plasma amino acids	Urea cycle defects (elevated Glu)
		MSUD (elevated branched-chain amino acids)
		Tetrahydrobiopterin deficiencies (elevated Phe)
		Lactic acidemias (elevated Ala)
		Pyruvate carboxylase deficiency (elevated Cit, Pro & Lys, low Glu)
		PNPO deficiency (high Gly and Thr)
		PDE (high Gly and Thr)
		Non-ketotic hyperglycinemia (elevated Gly)
		Hyperprolinemia type 2 (elevated Pro)
		Lipoic acid synthesis disorders (elevated Gly)
		Serine biosynthesis disorders (low Ser)
		Glutamine synthetase deficiency (low Gln)
		Asparagine synthetase deficiency (low Asn)
		GABA transaminase (elevated GABA, elevated beta-Ala)
		Mitochondrial glutamate transporter deficiency (elevated Pro)
		Molybdenum cofactor deficiency (low Cys, high Tau)
		Isolated sulfite oxidase deficiency (low Cys, high Tau)
	Plasma acylcarnitines	FAODs
		Organic acidemias
		Ethylmalonic encephalopathy
	Copper and ceruloplasmin	Menkes disease (low)
		Wilson disease (low)
	Plasma total homocysteine	Cobalamin C disease (high)
		MTHFR Deficiency (high)
		Molybdenum cofactor deficiency (low)
		Isolated sulfite oxidase deficiency (low)
URINE:	Urinalysis	Organic acidemias (elevated ketones)
		MSUD (elevated ketones)
	Urine AASA	PDE
		Molybdenum cofactor deficiency
		Isolated sulfite oxidase deficiency
	Urine purines and pyrimidines	Adenylosuccinate lyase deficiency (high succinyladenosine)
		Molybdenum cofactor deficiency (high xanthine and hypoxanthine)
	Creatine metabolites	AGAT deficiency (low GAA and creatine)
		GAMT deficiency (high GAA, low creatine)
		Creatine transporter deficiency (high creatine)
	Urine organic acids	PNPO deficiency (vanillactate)
		Organic acidurias
		OTC deficiency (orotic acid)
		Cobalamin C deficiency (MMA)
		Biotinidase deficiency and holocarboxylase synthetase deficiency (3-hydroxypropionic acid, 3-hydroxyisovaleric acid, 3-methylcrotonylglycine, methylcitrate)
		Fumarate hydratase deficiency (fumarate)
		SSADH deficiency (4-hydroxybutyric acid)
		Ethylmalonic encephalopathy (EMA)
		Mitochondrial short-chain enoyl-CoA hydratase 1 deficiency (methacrylylglycine, 3-hydroxyisobutyric acid, *S*−2-carboxypropyl-cysteine and *S*−2-carboxypropylcysteamine).

*AGAT, arginine:glycine amidinotransferase; CDG, congenital disorders of glycosylation; EMA, ethylmalonic acid; Fatty acid oxidation disorders, FAODs; GAA, guanidinoacetate; GAMT, guanidinoacetate methyltransferase; HHH, Hyperornithinemia-hyperammonemia-homocitrullinuria; HIHA, Hyperinsulinism-hyperammonemia syndrome; MSUD, maple syrup urine disease; MTHFR, methylenetetrahydrofolate reductase; PDE, pyridoxine-dependent epilepsy; PDH, pyruvate dehydrogenase; PNPO, pyridox(am)ine 5'-phosphate oxidase; SSADH, succinic semialdehyde dehydrogenase*.

##### Diagnostic therapies

Additionally, diagnostic therapy should be simultaneously applied in infants, which will supplement testing in the determination of etiology, and avoid therapeutic delay. For PDE, 100 mg of pyridoxine should be given intravenously (on day 1–3); samples can be collected after initiation of treatment. It is important to emphasize that this should be administered in a controlled setting (with resuscitation equipment available), since up to 20% of patients will develop cerebral depression, with the consequent risk of apnea. If responsive, then therapy should be continued with pyridoxine 15–30 mg/kg/day orally or enteral until diagnostic confirmation (elevated AASA in urine, blood and/or CSF and absence of urine sulfocysteine and at least 1 disease-causing mutation in the *ALDH7A1* gene). If seizures are unresponsive to pyridoxine during 3 or more days, then consider switching to pyridoxal 5′-phosphate (30 mg/kg/day orally divided in 4 doses) to treat PNPO deficiency. Patients who can swallow pills should do so, because aqueous PLP is subject to photodegradation, and these degradation products can activate hepatic stellate cells and might lead to hepatic complications (27). For younger children, the crushed tablet or content of the capsule should be dissolved immediately before administration, and the solution should be protected from light, to decrease the chance of photodegradation. Not all brands of PLP have the advertised amount of drug, with some brands have less or more content than advertised; in the UK PLP tablets from Solgar^Ⓡ^ were found to be the most reliable (28).

Alternatively, start with pyridoxine 15–30 mg/kg/day orally or enterally until results become available (if intravenous pyridoxine administration is not possible); clinical improvement of seizures is seen within minutes of intravenous pyridoxine administration, while EEG improvement can be delayed (seen within hours), and clinical improvement with oral pyridoxine can take 3–7 days to manifest. For biotinidase and holocarboxylase synthetase deficiencies, start infants on biotin 10 mg daily (until diagnosis is ruled out).

Folinic acid-responsive seizures have been shown to be of similar genetic origin to PDE (29); based upon this finding, we have decided not to include folinic acid in the initial diagnostic therapy step. Additionally, administration of folinic acid prior to extraction of a CSF sample may confound diagnostic accuracy by masking deficiency of methyltetrahydrofolate.

#### Tier 2: additional testing under the right clinical circumstances

Additional tests may be ordered by the clinician according to symptomatology and index of suspicion. This can be done in parallel to the 1st tier mentioned above, or sequentially. The list of these tests can be found in Table 4, along with the main clinical findings that might elicit suspicion of one of the treatable or non-treatable IEMs diagnosed with these tests.

**Table 4:** IEMs identified by Tier 2 diagnostic tests.

**Diagnostic test**	**Related treatable IEM**	**Related non-treatable IEM**
Blood smear		**Juvenile neuronal ceroid lipofuscinosis, GM1 gangliosidosis, Salla disease, fucosidosis** (vacuolated lymphocytes)
Carbohydrate-deficient transferrin		**Disorders of glycosylation**feat: Intellectual disability, ataxia, inverted nipples, abnormal fat pads.
Leukocyte CoQ10	**Primary CoQ**_10_ **deficiency** feat: Encephalomyopathy, nephrotic syndrome, hearing loss, cerebellar ataxia.
Blood Enzyme Activity: •Glucocerebrosidase •Arylsulfatase-A •Biotinidase •PPT1/TPP1	**Metachromatic leukodystrophy** feat: Dementia, psychosis/(manic-) depression, behavioral disturbance, spasticity, neuropathy. **Biotinidase deficiency** feat: Encephalopathic crisis, sensorineural hearing loss, eczema, alopecia.	**Gaucher disease type 3** feat: Dementia, horizontal supranuclear gaze palsy. Epilepsy.**Neuronal ceroid lipofucinosis** feat: accumulation of autofluorescent ceroid lipopigments, retinal pathology leading to blindness, dementia, epilepsy.
Very long chain fatty acids		**X-linked adrenoleukodystrophy**feat: cognitive deficits, progressive demyelination of CNS, visual loss, sensorineural deafness, convulsions and dementia.**Peroxisomal biogenesis disorder (Zellweger spectrum)**feat: developmental delay, hypotonia, vision problems, hearing loss, liver dysfunction, renal cysts, epiphyseal stippling, demyelination.
Urine oligosaccharides		**GM2 gangliosidosis (Tay-Sachs disease, Sandhoff disease)**feat: cherry-red spot, hypotonia, hepatosplenomegaly, ataxia, myoclonus, spastic tetraparesis, decerebration**Sialidosis**feat: Type I: cherry-red spot, myoclonus syndrome (responds to alcohol), gait abnormalities, epilepsy. Type II: progressive psychomotor decline, facial dysmorphism, myoclonus, kyphosis**Aspartylglucosiminuria**feat: slowly developing mental decline, beginning with clumsiness, late speech, hyperkinesia, mild facial dysmorphism, slight kyphoscoliosis
Urine sialic acid		**Salla disease**feat: Hypotonia, progressive neurodegeneration, hypomyelination.
Urine sulfocysteine		**Molybdenum cofactor and isolated sulfite oxidase deficiencies**feat: epileptic encephalopathy, lens dislocation, hyperekplexia, multicystic encephalomalacia.
CSF:plasma glucose ratio	**GLUT1 deficiency** feat: Ataxia, cerebral atrophy, epilepsy, non-epileptic movement disorder.
CSF lactate/pyruvate	**GLUT1 deficiency**	**Mitochondrial disorders**
CSF amino acids	**Serine biosynthesis disorders** feat: Epilepsy, neuropathy, cerebral atrophy, microcephaly, ichthyosis. Severe forms with Neu-Laxova syndrome.	**Nonketotic hyperglycinemia** feat: apnea, corpus callosum dysgenesis, epilepsy.**Glutamine synthetase deficiency**feat: epileptic encephalopathy, delayed gyration, necrolytic erythema**Asparagine synthetase deficiency**feat: epileptic encephalopathy, spastic quadriplegia, microcephaly, hyperekplexia.
CSF biogenic amines and BH4	**Tetrahydrobiopterin deficiencies** •**DHPR deficiency:** Developmental delay, epilepsy, microcephaly, Parkinsonism, cerebral atrophy and basal ganglia calcifications. •**AR GTPCH deficiency:** Developmental delay, epilepsy, microcephaly, truncal hypotonia, limb hypertonia. •**PTPS deficiency:** Developmental delay, epilepsy, microcephaly, Parkinsonian dystonia, lethargy, autonomic dysfunction. **Tyrosine Hydroxylase deficiency** feat: dystonia, postural tremor, developmental and motor delay, limb rigidity, hypokinesia, ptosis and oculogyric crisis.
	**AADC deficiency** Developmental delay, hypotonia, autonomic symptoms, non-epileptic movement disorder, oculogyric crises, epilepsy.	
CSF tetrahydrofolate	**FOLR1 deficiency** feat: psychomotor decline, epilepsy with drop attacks, progressive movement disorder, hypomyelination. **MTHFR deficiency** feat: developmental delay, hypotonia, apnea, epilepsy, thrombosis, brain atrophy. **DHFR deficiency** feat: megaloblastic anemia, seizures. **Serine biosynthesis disorders** feat: Microcephaly, developmental delay, spasticity, seizures, neuropathy, ichthyosis.

Given the large number of treatable IEMs presenting with epilepsy in infancy, and given that there are no clear clinical indicators that would set them apart from other epilepsy syndromes, we recommend that all infants presenting with epilepsy should undergo a lumbar puncture and CSF biochemical testing. In fact, the younger the infant, the more likely a lumbar puncture will be performed in order to rule out meningitis or viral encephalitis. It should be noted that the blood sample for glucose should always be obtained before the CSF sample is obtained, as otherwise the catecholaminergic response to the lumbar puncture would increase the serum glucose and alter the ratio. Similarly, the lumbar puncture should be collected while the patient is off intravenous dextrose, and fasting. Specific cutoffs for establishing the diagnosis of GLUT1 deficiency based on CSF glucose, CSF lactate, and CSF-to-blood glucose ratio have been published (30). Since the blood sample would be obtained before the lumbar puncture, a sample for plasma amino acids should be collected at the same time. For sampling of CSF neurotransmitters, it is important to note that a rostrocaudal gradient exists for metabolites derived from substances produced in the brain; this means that the more CSF that is drawn, the higher the values of HVA and 5HIAA will go–in fact, values can double with every 5 ml of CSF drawn (31). The reference ranges are established by each laboratory using a specific CSF fraction. Thus, clinicians should adhere to a sampling protocol with numbered tubes, following the specific instructions provided by the laboratory performing the test. Gradients also exist for certain amino acids, with GABA and taurine exhibiting a rostrocaudal gradient, while alanine and asparagine show a reverse gradient (32). Additionally, tetrahydrobiopterin (BH4) is extremely sensitive to oxygen and light, so the tube used to collect BH4 should contain antioxidants and stored in dark to protect the sample integrity. Finally, the CSF samples should be immediately frozen at the bedside. However, if the lumbar puncture was traumatic, blood cells should be removed by centrifugation (to avoid metabolite oxidation by products of hemolyzed RBCs), and only clear CSF should be frozen in new tubes (31). Although neurotransmitter analysis may still be possible, the serum component of the blood is not removed by centrifugation, and thus the CSF amino acid analysis would still have major artifacts in the setting of a traumatic lumbar puncture. Hence, a repeat lumbar puncture might be needed in this setting.

### Genomics, metabolomics, and multi-omics approaches

During the past few years, the field of genomics has seen the advent of faster and cheaper technologies that have revolutionized clinical care. In the field of epilepsy in particular, a recent paper described a diagnostic yield of 14% (49 diagnosed patients out of 349 patients with drug-resistant pediatric epilepsy) when 30 genes were sequenced, while expanding the sequencing panel to 95 genes increased the diagnostic yield to 20.3% (71/349 patients) (33). It is important that if any such epilepsy sequencing panel is offered, that all genes known to be associated with our list of treatable metabolic epilepsies be included, given the important management implications.

A recent study described a small cohort of 32 patients with epileptic encephalopathy who had undergone prior testing including neurologic and genetics consultations, metabolic screening, neuroimaging, neurophysiologic studies, chromosomal microarray, and targeted genetic testing. In this cohort, the diagnostic yield of whole exome sequencing was 50% (16/32 patients), and it was the most cost-effective way to reach a diagnosis, with a cost approximately 10 times less than a standard diagnostic approach (34). There are, however, limitations to the use of either a sequencing panel or whole exome sequencing. As an example, a recent study integrated alternative transcripts for known neonatal epilepsy genes with RNA-Seq, and found that in 30% of cases (89 brain-expressed alternative coding regions in 292 neonatal epilepsy genes) the alternative transcripts corresponded to a noncoding segment in the canonical transcript analyzed by standard clinical tests (35). This is important because in some cases, pathogenic variants were found in these non-canonical brain transcripts; furthermore, half to two-thirds of these alternative coding regions are captured by common exon capture kits, even though these normally fall out of the scope of analysis. Another limitation of the next-generation sequencing approach is that in general the turnaround time for results is longer than for the standard biochemical tests mentioned so far; thus, the biochemical workup for treatable etiologies of metabolic epilepsies should not be postponed, and should occur previously to or concurrently with the genomic workup. Also, the results can be complimentary, with biochemical findings confirming or ruling out the functional effect of variants of unknown significance, while in other cases with negative exome results, biochemical abnormalities may point to the perturbed pathway enabling a targeted or manual inspection of candidate genes for any missed or cryptic variants.

Another diagnostic possibility available in this day and age is that of metabolomics, which is widely available on a research basis, but is only offered on a clinical basis by a single laboratory in the US. In this laboratory, the use of clinical metabolomics led to the diagnosis of three patients with GABA transaminase deficiency, siblings with L-2-hydroxyglutaric aciduria, one patient with aromatic amino acid decarboxylase deficiency, and one patient with adenylosuccinase deficiency in a single year (36). In addition to diagnostics, the use of metabolomics and the combined approach of genomics and metabolomics approaches can lead to the identification of novel biomarkers, and can be used to confirm pathogenicity of variants of unknown significance (37).

### Referral to metabolic diseases specialist

For any patient who shows abnormalities on any of the tier 1 or 2 metabolic tests (even before confirmation of an IEM), the biochemical physician on-call should be contacted as soon as possible for further diagnostic testing and management. Other features that should prompt referral to a metabolic specialist include

- Progressive symptoms suggestive of neurodegeneration
- Developmental regression or plateauing
- Significant behavioral deterioration from baseline pattern
- Refractory seizures
- Unexplained movement disorder
- MRI/S of the brain showing unexplained abnormalities
- Recurrent/unexplained emesis
- Coarse facial features
- Hepato- and/or splenomegaly
- X-ray evidence of skeletal dysplasia
- Documented episodes of:
  - Hypoglycemia
  - Significant metabolic acidosis
  - Ketonuria (unusual for clinical circumstances).

**Figure 1:**
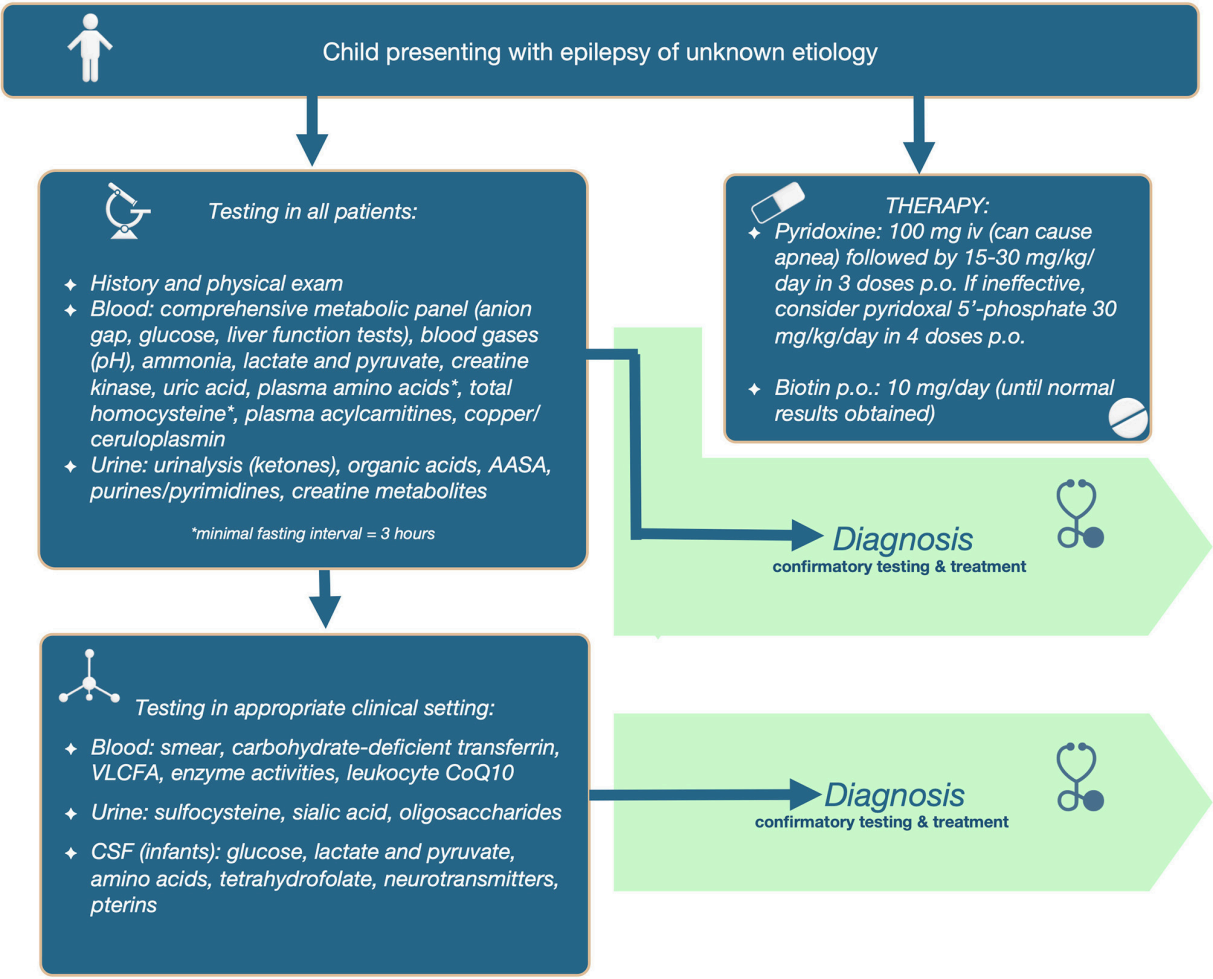
Diagnostic algorithm for metabolic epilepsies.

The full diagnostic algorithm can be found in Figure 1.

## Conclusion

Based on a literature review, we provide an updated algorithm for the diagnosis of metabolic epilepsies, taking into account the description of novel IEMs in recent years up to 2018. The clinician's expertise and insights into the patient's clinical presentation remain paramount in the diagnostic work-up, as reflected in our algorithm. In fact, for several IEMs, treatment should be started even before confirmation of diagnosis. Our protocol includes tests chosen according to amenability to treatment of the IEMs potentially identified by the test, diagnostic yield, availability, and affordability. This algorithm is meant to provide a structure to the diagnostic workup, but does not claim to be exhaustive. In fact, the current protocol should be regularly updated in order to accommodate newly discovered IEMs and treatments in the future.

To conclude, this protocol has been developed as a standardized testing procedure to increase diagnostic efficacy of those IEMs amenable to treatment as well as reduce costs and diagnostic delay. While this protocol has been organized based upon literature and clinical observations, it is ultimately reliant upon the attending clinician's best judgment to implement an optimal investigative plan according to established parameters. In fact, the current protocol should be regularly updated in order to accommodate newly discovery IEMs, such as uridine-responsive encephalopathy due to biallelic CAD mutations and vitamin B6-responsive epileptic encephalopathy due to biallelic PLPBP mutations (38, 39). Aside from exome sequencing, entry into the clinical arena of integrated multi-omics analyses such as metabolomics and transcriptomics will certainly catalyze such discoveries further (40). Finally, approval of novel treatments such as gene therapy for metachromatic leukodystrophy (41, 42) will require regular updates of the current protocol, and this is favorable news for our patients and families.

## Author contributions

CvK and GH designed the study. LT, JL, and BS collected data. CvK, GH, and CF wrote the manuscript and designed the algorithm. All authors contributed the lit review, edited and approved manuscript for publication.

### Conflict of interest statement

The authors declare that the research was conducted in the absence of any commercial or financial relationships that could be construed as a potential conflict of interest.

## Supplementary material

Supplementary Table 1 Comprehensive list of “metabolic epilepsies,” defined as inborn errors of metabolism presenting with epilepsy.
